# VEGF-A-Cleavage by FSAP and Inhibition of Neo-Vascularization

**DOI:** 10.3390/cells8111396

**Published:** 2019-11-06

**Authors:** Özgür Uslu, Joerg Herold, Sandip M. Kanse

**Affiliations:** 1 Institute for Biochemistry, Justus-Liebig-University Giessen, 35392 Giessen, Germany; Oezguer1975@gmx.de; 2 Department of Angiology, Clinic for Vascular Medicine, Klinikum Darmstadt, 64283 Darmstadt, Germany; joerg_herold@hotmail.com; 3 Institute for Basic Medical Sciences, University of Oslo, Sognvannsveien 9, 0372 Oslo, Norway

**Keywords:** factor VII activating protease, HABP2, VEGF, matrigel, neo-vascularization, hind limb ischemia

## Abstract

Alternative splicing leads to the secretion of multiple forms of vascular endothelial growth factor-A (VEGF-A) that differ in their activity profiles with respect to neovascularization. FSAP (factor VII activating protease) is the zymogen form of a plasma protease that is activated (FSAPa) upon tissue injury via the release of histones. The purpose of the study was to determine if FSAPa regulates VEGF-A activity in vitro and in vivo. FSAP bound to VEGF_165_, but not VEGF_121_, and VEGF_165_ was cleaved in its neuropilin/proteoglycan binding domain. VEGF_165_ cleavage did not alter its binding to VEGF receptors but diminished its binding to neuropilin. The stimulatory effects of VEGF_165_ on endothelial cell proliferation, migration, and signal transduction were not altered by FSAP. Similarly, proliferation of VEGF receptor-expressing BAF3 cells, in response to VEGF_165_, was not modulated by FSAP. In the mouse matrigel model of angiogenesis, FSAP decreased the ability of VEGF_165_, basic fibroblast growth factor (bFGF), and their combination, to induce neovascularization. Lack of endogenous FSAP in mice did not influence neovascularization. Thus, FSAP inhibited VEGF_165_-mediated angiogenesis in the matrigel model in vivo, where VEGF’s interaction with the matrix and its diffusion are important.

## 1. Introduction

FSAP (factor VII activating protease) is a serine protease that circulates in plasma as an inactive zymogen. It belongs to the family of proteases that also includes the urokinase-plasminogen activator (uPA), tissue-PA (tPA), as well as hepatocyte growth factor activator (HGFA). Although a number of charged molecules can activate FSAP into the active protease (FSAPa), histones are the only endogenous molecules identified so far that can activate the zymogen form into FSAPa in plasma and in vivo [1]. In situations such as tissue injury [2], apoptosis, or necrosis [3], as well as when neutrophils undergo NETosis [4], the DNase activity in blood [5] is likely to release histones. FSAPa in turn can cleave and degrade histones and decrease their toxicity towards cells [3,4]. A single nucleotide polymorphism (SNP) in the FSAP gene, Marburg I (MI, G534E) is associated with a weak proteolytic activity [6] and an increased risk of carotid stenosis [7], stroke [8], venous thrombosis [9,10], liver fibrosis [11], and thyroid cancer [12]. The relationship to venous thrombosis [13] and thyroid cancer [14] was not replicated in a number of subsequent studies. 

This relationship between the loss of FSAP activity and diseases is also replicated in FSAP-deficient *(Habp2^-/-^*) mice*. Habp2^-/-^* mice show no explicit characteristics when maintained under standard pathogen-free laboratory conditions and do not exhibit any developmental abnormalities. These mice have been studied in two different models of vascular remodeling. In the wire-induced injury model of neointima formation, *Habp2^-/-^* mice formed a bigger neointima than wildtype (WT mice) [15]. In the model of hind limb ischemia, arteriogenesis in the adductor muscle was enhanced in *Habp2^-/-^* mice, whereas neovascularization was unchanged in the gastrocnemius muscle [16]. Thus, the lack of *Habp2* gene in mice promotes a more exacerbated repair response that is related to enhanced inflammation and increased activity of the pericellular proteolysis system [15,16]. 

The effects of FSAP in relation to human diseases and mouse models is likely to be related to proteolysis of different substrates. Although a number of substrates for FSAP have been identified [17] we will focus here only on pathways that are linked to vascular remodeling. Growth factors are cleaved by FSAP, which in some cases leads to a loss of activity, such as platelet derived growth factor-BB (PDGF-BB) [18]. PDGF-BB cleavage leads to an inhibition of vascular smooth muscle cells (VSMC) migration and proliferation, as well as neointima formation. FSAP inhibits basic fibroblast growth factor (bFGF)-mediated endothelial cell proliferation by binding to and/or slowly degrading the growth factor [19] and can also activate bFGF by releasing it from the matrix [20]. Activation of bone morphogenetic protein (BMP)-2 and the conversion of pro-BMP-2 into the active form of cytokine is also a function of FSAP that leads to differentiation of cells [21]. FSAP also cleaves protease activated receptors (PARs)-1 and -3 and influences vascular permeability in combination with hyaluronic fragments of different molecular weights [22]. PAR-1 was identified as a receptor on astrocytes and neurons that mediate the anti-apoptotic effects of FSAP in the context of stroke [23]. Stimulation of VSMC and endothelial cells by FSAP leads to an increased expression of proinflammatory genes in both cells types. Whereas the effect of FSAP could be clearly ascribed to PAR-1 on VSMC, this was clearly not the case for endothelial cells. 

Vascular endothelial growth factor (VEGF) is a key factor for determining endothelial lineage, endothelial cell proliferation and migration, as well as recruitment of pericytes and vessel assembly [24]. It belongs to the cysteine knot family of growth factors that include the four genes of the PDGF family as well as placental growth factor (PLGF). Of the four genes encoding for VEGF, denoted A, B, C, and D, VEGF-A is considered to be the most important for hypoxia-driven angiogenesis and is secreted in multiple forms, such as VEGF_121_, VEGF_165_, and VEGF_189_, by alternative splicing [25]. These isoforms have a common N-terminal region for receptor binding, whereas the C-terminal part that mediates binding to co-receptors such as neuropilin and cell- and matrix-associated proteoglycans (ECM) [26] is progressively longer. This C-terminal region has a cluster of negatively charged amino acids and has cleavage sites for uPA, plasmin, and matrix metalloproteinases [27], which regulate VEGF’s association with the matrix and co-receptors and results in a different pattern of neovascularization. 

With the knowledge that FSAP can cleave proteins at clusters of basic amino acids [17] and that it cleaves PDGF-BB [18], we hypothesized that the homologous protein VEGF-A is also cleaved, and its activity regulated by FSAP. We performed binding and cleavage studies with purified proteins to show that FSAP can indeed cleave long forms of VEGF in their heparin/neuropilin-binding domain and that, as expected, this disturbs their binding properties. However, no modulation of VEGF_165_ activity in vitro on cellular functions was observed. An inhibiting effect of FSAP in the in vivo matrigel model of neovascularization model was observed, which supports the notion that this effect of FSAP may operate in vivo where matrix association, sequestration, and release of VEGF are decisive. 

## 2. Material and Methods

*FSAP preparations:* The isolation of wild type-FSAP as well as the MI-SNP (G534E isoform) from human plasma, along with the preparation of enzymatically inactivated Phe-Pro-Arg-chloromethylketone (PPACK)-FSAP has been described before [18,28]. The buffer for storage of FSAP was 0.2 M arginine, 0.2 M lysine, 5 mM citrate, pH 4.5, and was also used at the appropriate dilution to exclude any influence of the vehicle. Because of the rapid auto-activation of the zymogen form of FSAP into FSAPa, under the experimental conditions used in this study, the term FSAP is synonymous for FSAPa.

*Specific cleavage of VEGF isoforms:* VEGF_165_ or VEGF_121_ (R & D Systems, Wiesbaden, Germany) (2 µg/mL) was incubated with FSAP (12 µg/mL) in Tris pH 7.4, 100 mM NaCl, 2 mM CaCl_2_ for 1 h at 37 °C in the absence or presence of heparin (10 µg/mL) or aprotinin (15 µg/mL), and the reaction was stopped with SDS sample buffer. Western blots were performed under non-reducing and reducing conditions (β-mercaptoethanol; 10%, vol/vol) and VEGF was detected with a polyclonal goat antibody from R & D Systems. Cleaved VEGF was subjected to amino terminal sequencing using the automated Edman degradation procedure with an online phenylthiohydantoin derivative analyzer (Applied Biosystems, Darmstadt, Germany). 

*Binding interactions between FSAP, VEGFR2, VEGF, and neuropilin:* VEGF_121_ or VEGF_165_ was immobilized in a Maxisorp microtiter 96-well plate (Nunc, Roskilde, Denmark) at a concentration of 1 µg/mL (50 mL) overnight at 4 °C in 50 mM NaHCO_3_ buffer, pH 9.6. The plate was blocked with 3% (wt/vol) BSA in Tris pH 7.4, 100 mM NaCl. FSAP (0–2 µg/mL) was added to the wells with 0.3% (wt/vol) BSA for 2 h at 22 °C. After extensive washing, bound FSAP was detected with an antibody followed by peroxidase-linked secondary antibody. The binding of ligands to BSA-coated wells was used as a blank in all the experiments and was subtracted to obtain specific binding. Similarly, either neuropilin-1-Fc or VEGFR2-Fc (R & D Systems) were immobilized to study the binding of FSAP or VEGF. 

*Cellular assays*: Human umbilical vein endothelial cells (HUVEC) were cultivated in ECBM medium (modified MCDB-151) containing 5% (vol/vol) FCS (Promocell, Heidelberg, Germany) on fibronectin-coated dishes. For regular growth of these cells, the medium was supplemented with amphotericin B (50 ng/mL), gentamicin (50 ng/mL), epidermal growth factor (0.1 ng/mL), and bFGF (1.0 ng/mL), as described by the manufacturer. Growth factors were preincubated with FSAP for 60 min at 37 °C before stimulation of serum starved cells, as described previously [18]. DNA synthesis was determined using the BrdU incorporation kit from Roche Diagnostics (Mannheim, Germany). Migration was tested in a Boyden chamber on a collagen type I coated membrane with 8 µm pores. Growth factors were preincubated with FSAP for 60 min at 37 °C before cell stimulation. Cells were incubated in medium containing 0.1% (vol/vol) FCS in the upper chamber, whereas the lower chamber received the same medium with different additives, as indicated. After an incubation period of 5 h at 37 °C, the upper side of the membrane was scraped to remove all cells. Thereafter, the membrane was fixed, stained, and the optical density of each well was measured to quantify cell migration. Cells were lysed in SDS-sample buffer and applied onto SDS-PAGE followed by western blotting and detection of phosphorylated ERK with a phospho-specific antibody with total ERK as a loading control (both from Cell Signaling Technology, Leiden, The Netherlands). 

BAF3-VEGFR2 cells were obtained from Steven Stacker and Marc Achen (Ludwig Cancer Research Institute, Melbourne Branch, Australia) and BAF3-VEGFR1 cells were provided by Kari Alitalo (Ludwig Cancer Research Institute, Helsinki, Finland) and were cultured in RPMI-1640 medium containing murine interleukin (IL)-3 (Strathman Biotech, Hannover, Germany). Cell number was determined by the WST-1 assay (Roche Diagnostics).

*Western blotting analysis*: HUVEC were starved for 4 h in serum-free medium and then stimulated for 15 min with the appropriate agonist. Cells were pre-incubated with inhibitors for 30 min before induction with agonist. The experiments were stopped by adding SDS sample buffer containing 10 mM NaF, 1 mM orthovanadate, and 1 mM pyrophosphate, and the samples were processed for western blotting. SDS-PAGE was performed and proteins were transferred to Hybond nitrocellulose membranes (GE Healthcare, Freiberg, Germany). For analysis of western blotting, ECL prime chemiluminescence (GE Healthcare) was used. Tissue pieces were homogenized in a glass homogenizer in TBS (50 mM Tris, pH 7.4, containing 100 mM NaCl) with 1% (w/v) SDS. After centrifugation, the extracts were frozen at −80 °C until further analysis. Densitometric analysis was performed to calculate relative expression using ImageJ (NIH, Bethesda, Maryland, USA).

*Matrigel model of* in vivo *angiogenesis*: The matrigel model was performed essentially as described previously [29] and there were 7–8 mice per group. Growth factor-reduced matrigel (BD Biosciences) was supplemented with heparin (200 μg/mL), VEGF_165_, and bFGF (200 ng/mL each), FSAP, MI-FSAP, or PPACK-FSAP (12 μg/mL), as well as the appropriate volume of buffer control. Without the presence of heparin, the neovascularization response is very weak in this model system. The concentration of each growth factor was halved when used in combination. Matrigel was applied subcutaneously into the right and left underside flank of 8–12 week old female C57/BL6 mice. The mice were sacrificed after 7 days and the matrigel plugs were removed, fixed with formaldehyde, and embedded in paraffin. Then, 7 µm serial sections were cut and mounted on slides, deparaffinized in xylene, and rehydrated through graded ethanol washes. After antigen retrieval with Tris-EDTA buffer (pH 9.0), the sections were stained with endothelial-specific lectin called bandeirea simplificifolia-1 (BS-1, FITC labelled) [30] and Cy3 labelled anti-α-SMA (α smooth muscle specific-actin) (Sigma) and DAPI. The number of red/green positive vessels counted per section from three different levels of the matrigel plugs and vascular density was expressed as number of vessels per mm^2^. Percentage area of anti-α SMA, BS-1, and DAPI staining was also quantified using ImageJ. All three parameters essentially gave qualitatively very similar results, and only bona-fide vessel density results are presented here. We also localized von Willebrand factor (vWF), an alternative marker for endothelial cells (rabbit polyclonal, DAKO, Glostrup, Denmark), in some staining experiments. Both methods of endothelial cell quantification gave similar results. 

*Hind limb ischemia model and western blotting of VEGF-A:* The model of hind limb ischemia and the resulting changes in arteriogenesis and angiogenesis have been described in detail in our earlier manuscript [16]. Western blotting with anti-VEGF-A and anti-COX IV was performed to adjust for differences in protein concentrations. Band density was measured using the image analysis system Quantity One system (Biorad, Munich, Germany). Experiments were performed in four mice per group.

*Statistics and reproducibility:* All biochemical and cellular experiments were performed in 3-5 independent experiments. Statistical significance was tested by using analysis of variance (ANOVA) with Bonferroni post-hoc test using the programme Graphpad Prism Biostatistics Software (Graphpad, San Diego, CA). A *p*-value < 0.05 was considered to be significant. 

*Study approval:* All procedures involving experimental animals were approved by the local government animal care committee (GI 20/10-No. 66/2012) and complied with the Directive 2010/63/EU of the European Parliament.

## 3. Results

**Figure 1:**
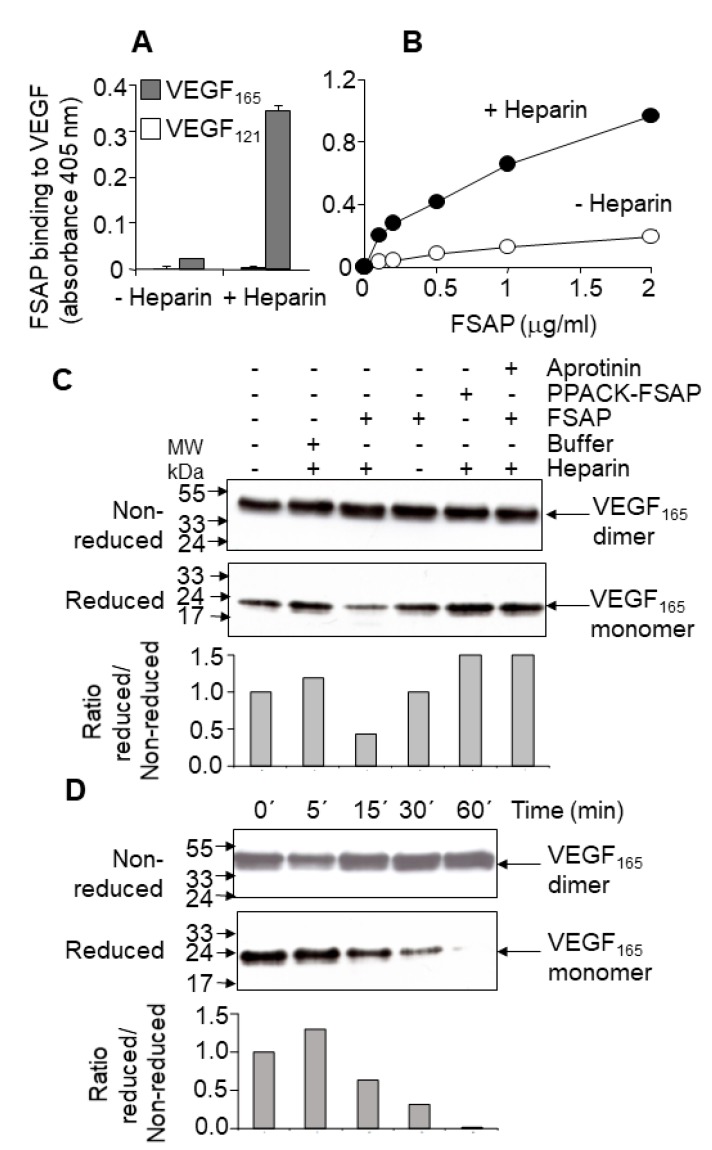
Binding of FSAP (factor VII activating protease) to VEGF (vascular endothelial growth factor) and its specific proteolytic cleavage: (**A**) VEGF_121_ (open bars) or VEGF_165_ (grey bars) was immobilized, and the binding of FSAP, in the absence or presence of heparin, was detected with an anti-FSAP antibody (mean ± SD of triplicate wells); (**B**) VEGF_165_ was immobilized, and the binding of increasing concentrations of FSAP in the absence (open circles) or presence (filled circles) of heparin was determined. Error bars are smaller than the size of the symbols; (**C**) Mixtures of FSAP (or Phe-Pro-Arg-chloromethylketone (PPACK)-FSAP), buffer, heparin, VEGF_165_, and aprotinin, as indicated, were incubated, and the reaction was analyzed by western blotting with an anti-VEGF antibody under reducing or non-reducing conditions; (**D**) FSAP, VEGF_165_, and heparin were incubated for the indicated time intervals and the samples were analyzed for VEGF, as above. Densiometric analysis was performed to calculate the ratio of VEGF under reduced and non-reduced conditions.

**Figure 2:**
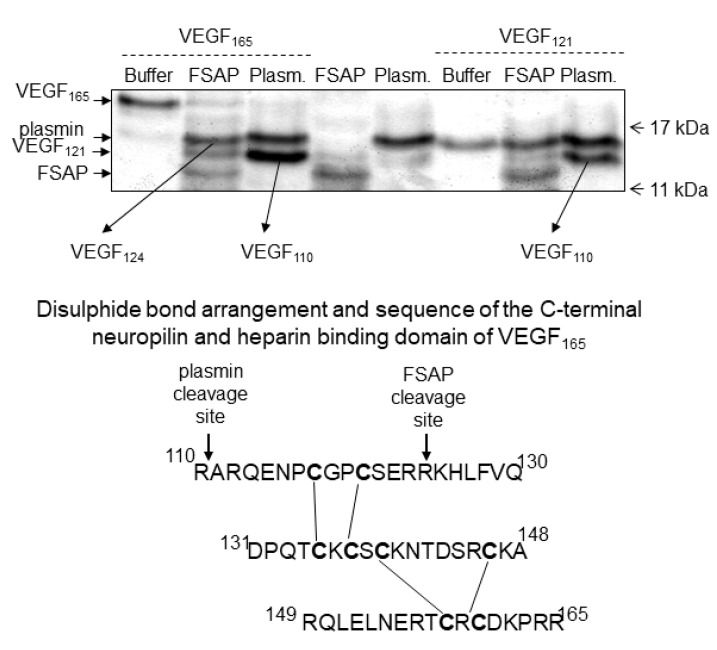
Sequencing of proteolytically cleaved VEGF: VEGF_165_ or VEGF_121_ (20 μg/mL) was incubated with FSAP (200 μg/mL) or plasmin (200 μg/mL) in the presence of heparin (10 μg/mL) for 2 h at 37 °C. The mixture was separated by SDS-PAGE under reducing conditions and processed for N-terminal sequencing. The FSAP and plasmin cleavage sites, as well as the disulphide bond assignments in the heparin/neuropilin-binding domain [31] sequence of VEGF_165_ are indicated.

*Binding and cleavage of VEGF-A by FSAP:* In solid phase binding assays, FSAP bound to immobilized VEGF_165_, but not to VEGF_121_, in the presence of heparin (Figure 1A,B). Under non-reducing conditions, there was no change in the intensity or size of the VEGF_165_ upon incubation with FSAP (Figure 1C,D); however, under reducing conditions, there was a decrease in VEGF immunoreactivity (Figure 1C,D). Cleavage was enhanced by heparin, which often functions as a co-factor for FSAP activity. Inhibition of FSAP by aprotinin reduced this cleavage. The enzymatically inactive PPACK-FSAP had no effect (Figure 1C). Cleavage of VEGF_165_ was time-dependent (Figure 1D), but VEGF_121_ was not cleaved at all (Supplementary Figure S1). The cleavage site was identified by amino acid sequencing and was found to be in the neuropilin/heparin-binding domain at position 124/125, which was distinct from the plasmin cleavage site at 110/111 (Figure 2). The cleavage site for plasmin and FSAP in relation to the disulphide bridges [31] is shown in Figure 2. Thus, FSAP cleaves VEGF_165_ in the heparin/neuropilin-binding region. 

**Figure 3:**
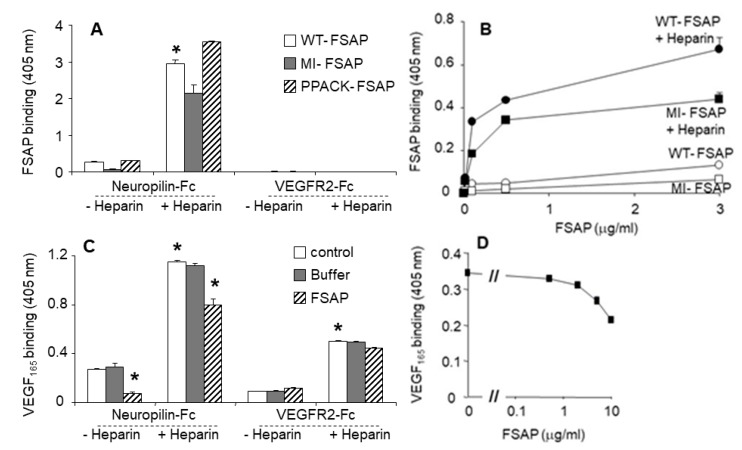
Interactions between FSAP, neuropilin, VEGFR2, and VEGF_165_: (**A**) Neuropilin-1-Fc or VEGFR2-Fc was immobilized and the binding of wild type (WT)-FSAP (open bars), Marburg I (MI)-FSAP (G534E-SNP) (grey bars), or PPACK-FSAP (hatched bars) was determined in the absence or presence of heparin; (**B**) To immobilized neuropilin-1-Fc, increasing concentrations of WT-FSAP (circles) or MI-FSAP (squares) in the absence (open symbols) or presence (closed symbols) of heparin (filled circles) was added, and FSAP binding was determined; (**C**) Neuropilin-1-Fc or VEGFR2-Fc was immobilized, and the binding of VEGF_165_ was determined in the absence or presence of heparin (open bars), buffer (grey bars), or FSAP (hatched bars); (**D**) Neuropilin-1-Fc was immobilized and the binding of VEGF_165_ was determined in the presence of heparin and increasing concentrations of FSAP, as indicated. Results are shown as absorbance (mean + SD of triplicate wells). Error bars in 2B and 2D are smaller than the size of the symbols. * *p* < 0.05.

*Interaction of FSAP with neuropilin and VEGFR-2:* Neuropilins are co-receptors that bind to larger VEGF isoforms, such as VEGF_165_, in a heparin-dependent manner, and regulate the activity of long forms of VEGF. Thus, the binding interactions between FSAP, VEGF_165_, VEGFR, and neuropilin were investigated. FSAP bound strongly to neuropilin-1-Fc in a heparin-dependent manner, but there was no binding to VEGFR-2-Fc (Figure 3A). There was no difference in the binding of PPACK-FSAP, the MI-SNP of FSAP, or WT-FSAP (Figure 3A,B), indicating that the enzymatic activity of FSAP was not involved in the binding to neuropilin. VEGF_165_ bound to both neuropilin and VEGFR2 in a heparin-dependent manner (Figure 3C). VEGF_165_ binding to neuropilin-1-Fc was partially inhibited by FSAP in the absence or presence of heparin, but FSAP had no influence on binding of VEGF_165_ to VEGFR-2-Fc (Figure 3C,D). Hence, FSAP binds to neuropilin, thereby cleaving VEGF_165_ that, in turn, partially decreases its interactions with neuropilin but not VEGFR2.

**Figure 4:**
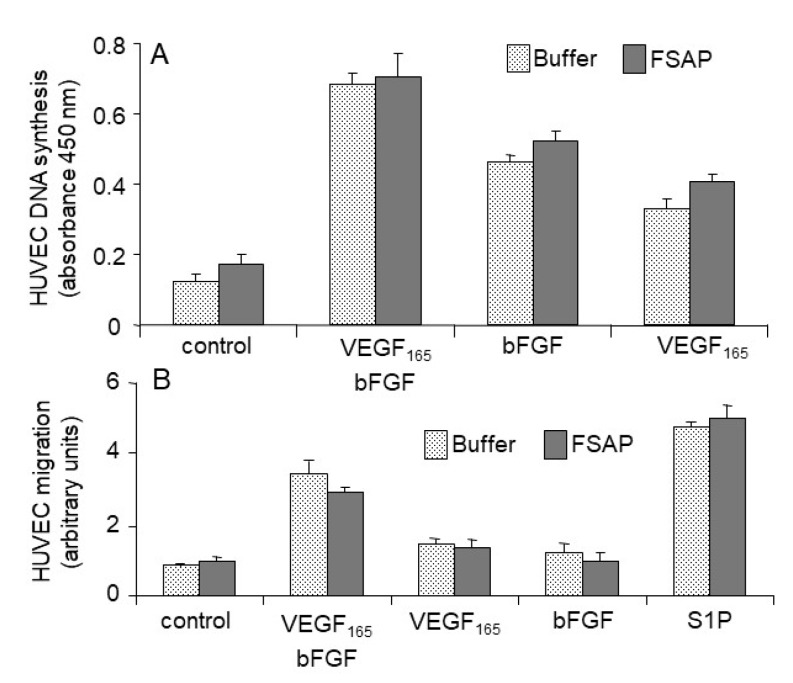
Effect of FSAP on proliferation and migration of human umbilical vein endothelial cells (HUVEC): basic fibroblast growth factor (bFGF) (40 ng/mL) and/or VEGF_165_ (20 ng/mL) in the presence of FSAP (12 µg/mL) (dark bars) or buffer control (dotted bars), as well as heparin (10 µg/mL) was preincubated for 60 min at 37 °C, and the mixtures were used to stimulate serum-starved HUVEC. (**A**) DNA synthesis was determined using the BrdU incorporation kit; (**B**) Migration was tested in a Boyden chamber. Sphingosine-1-phosphate (S1P) was used a positive control and its concentration was 200 nM. Data are mean + SD of triplicate wells.

**Figure 5:**
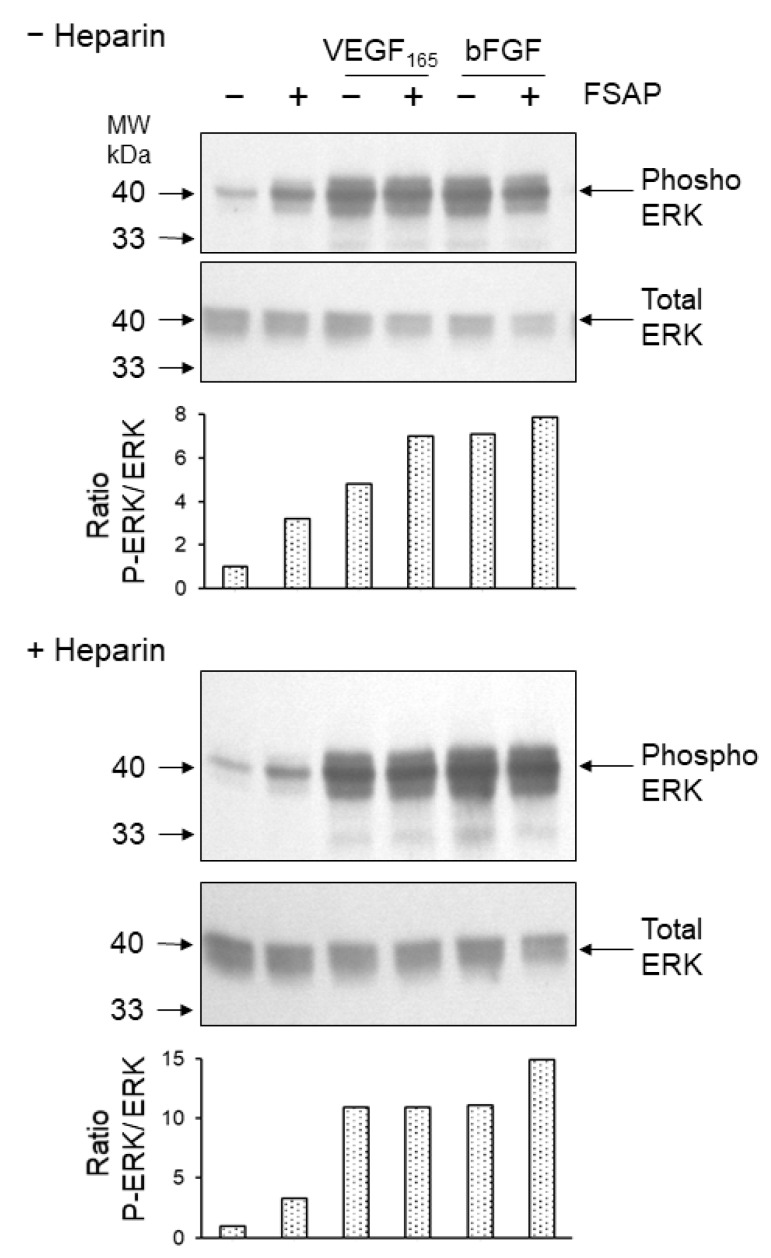
Effect of FSAP on ERK phosphorylation in HUVEC: Mixtures of FSAP (12 µg/mL), buffer, heparin (10 µg/mL), VEGF_165_ (20 ng/mL), and/or (bFGF 50 ng/mL) were preincubated for 1 h at 37 °C in serum-free medium and then added to cells for 15 min. Cells extracts were analyzed by Western blotting for phosphorylated ERK. Analysis of total ERK was performed to confirm equal loading of gel with lysates. Relative phospho ERK levels were determined by densiometric analysis.

*Effect of FSAP on proliferation and migration of HUVEC or VEGFR-transfected BAF3 cells:* Because FSAP can cleave VEGF_165_ and inhibits its binding to neuropilin, the effect of FSAP-treated VEGF_165_ on the activation of HUVEC was investigated in the absence or presence of heparin. FSAP did not influence bFGF- or VEGF_165_-induced DNA synthesis or cell migration (Figure 4A,B). Phosphorylation of ERK in HUVEC with bFGF or VEGF pretreated with FSAP was not altered (Figure 5). 

**Figure 6:**
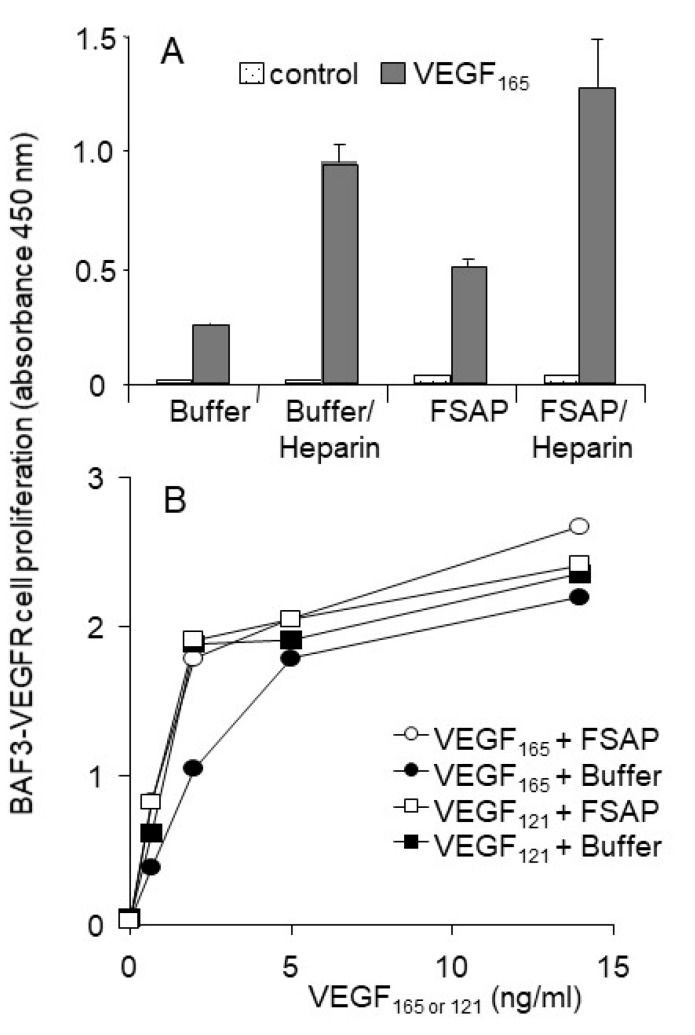
Effect of FSAP on VEGF-mediated proliferation of VEGFR expressing BAF3 cells: (**A**) VEGFR2-BAF3 cells were stimulated for 4 days with (dark bars) or without VEGF_165_ (10 ng/mL) (dotted bars) in the absence or presence of FSAP (12 μg/mL), as well as heparin (10 μg/mL); (**B**) VEGFR1-BAF3 cells were stimulated with VEGF_165_ (circles) or with VEGF_121_ (squares) in the absence (filled) or presence (open) of FSAP (12 μg/mL), as well as heparin (10 μg/mL). Cell number was determined by the WST-1 assay. Mean + SD of triplicate wells is shown.

In order to further characterize this result, we also tested the activation of VEGFR-transfected BAF3 cells that are very sensitive to the effects of VEGF. Even in this very sensitive cellular system, FSAP did not inhibit VEGF_165_- or VEGF_121_-induced proliferation of BAF-3 cells transfected with VEGFR1 or VEGFR2 (Figure 6A,B). FSAP did not inhibit the effect of bFGF on HUVEC, which is in accordance with our previous results on VSMC [18], but is in contrast to earlier studies on HUVEC [19,20,32]. Expression of neuropilin-1 was observed on both cell types by flow cytometry (data not shown). Thus, in different cellular test systems, FSAP-mediated cleavage of VEGF_165_ did not alter its ability to activate cellular functions.

**Figure 7:**
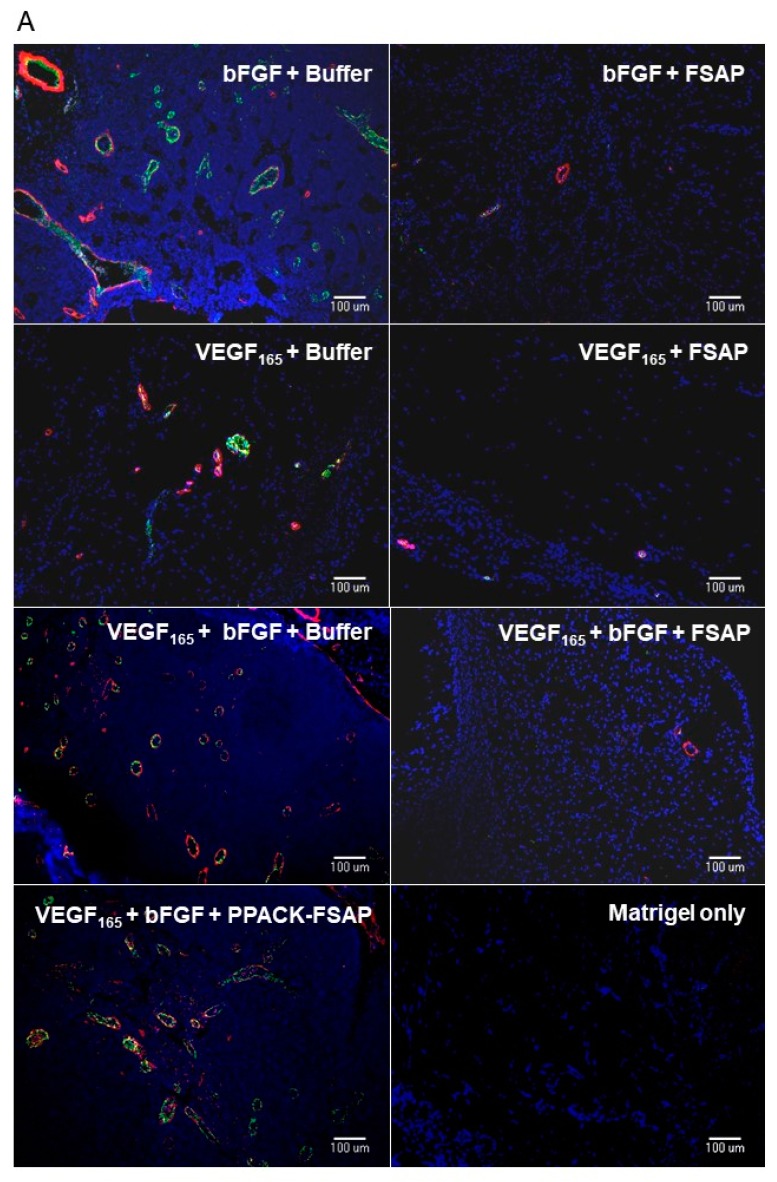

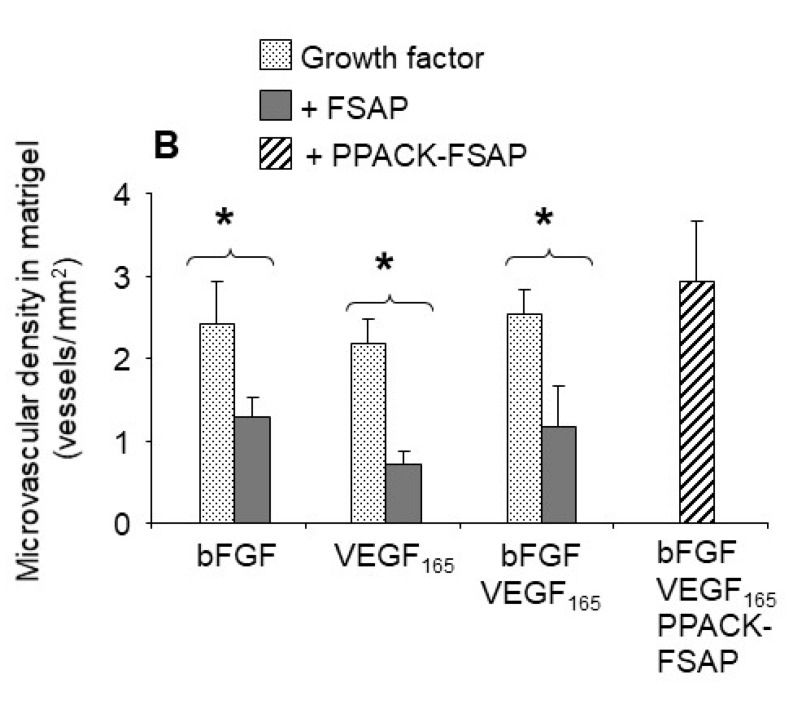
Effect of FSAP on microvascular density in matrigel plugs in vivo: (**A**) Photomicrographs of matrigel plugs after 7 days and stained for BS-1 (FITC, green), α-SMA (α smooth muscle specific-actin) (Cy3, red), and nuclei (DAPI, blue); (**B**) Microvascular density of plugs was determined, bars are means ± SEM (*n* = 7–8), * *p* < 0.05. Matrigel was supplemented with heparin and with either buffer (dotted bars), FSAP (black bars), or PPACK-FSAP (striped bars), as well as VEGF_165_ or bFGF, as indicated.

*Effect of FSAP on growth factor-mediated neo-vascularization in matrigel plugs in vivo:* We then tested the effect of FSAP in a model system where VEGF_165_ interaction with the matrix is important. This model was based on measuring neovascularization in vivo into matrigel plugs that is essentially an extract of tumor extracellular matrix. VEGF_165_ or bFGF, alone or in combination in the presence of heparin, stimulated the development of new vessels in matrigel, as determined by immunostaining for endothelial and smooth muscle cell markers (Figure 7A; endothelial-specific BS-1 (green) and α-SMA (α smooth muscle specific-actin) (red)). Quantification of the staining showed that the concomitant presence of FSAP reduced neovascularization induced by growth factors (Figure 7B). Enzymatically inactivated PPACK-FSAP did not inhibit growth factor-mediated neo-vascularization (Figure 7A,B), indicating the importance of the FSAP proteolytic activity for this effect. Similarly, the enzymatically inactive MI-isoform of FSAP did not inhibit VEGF_165_/bFGF-mediated neo-vascularization (Supplementary Figure S2). The effect of VEGF_121_-mediated neo-vascularization was not inhibited by FSAP (Supplementary Figure S2). Thus, exogenously applied FSAP could inhibit the effects of VEGF_165_, bFGF, and their combination on neovascularization in matrigel plugs in vivo. Preliminary experiments showed that the neovascularization into matrigel in response to growth factors, in the absence of endogenous FSAP *(Habp2^-/-^* mice), was similar as in WT mice (data not shown). 

**Figure 8:**
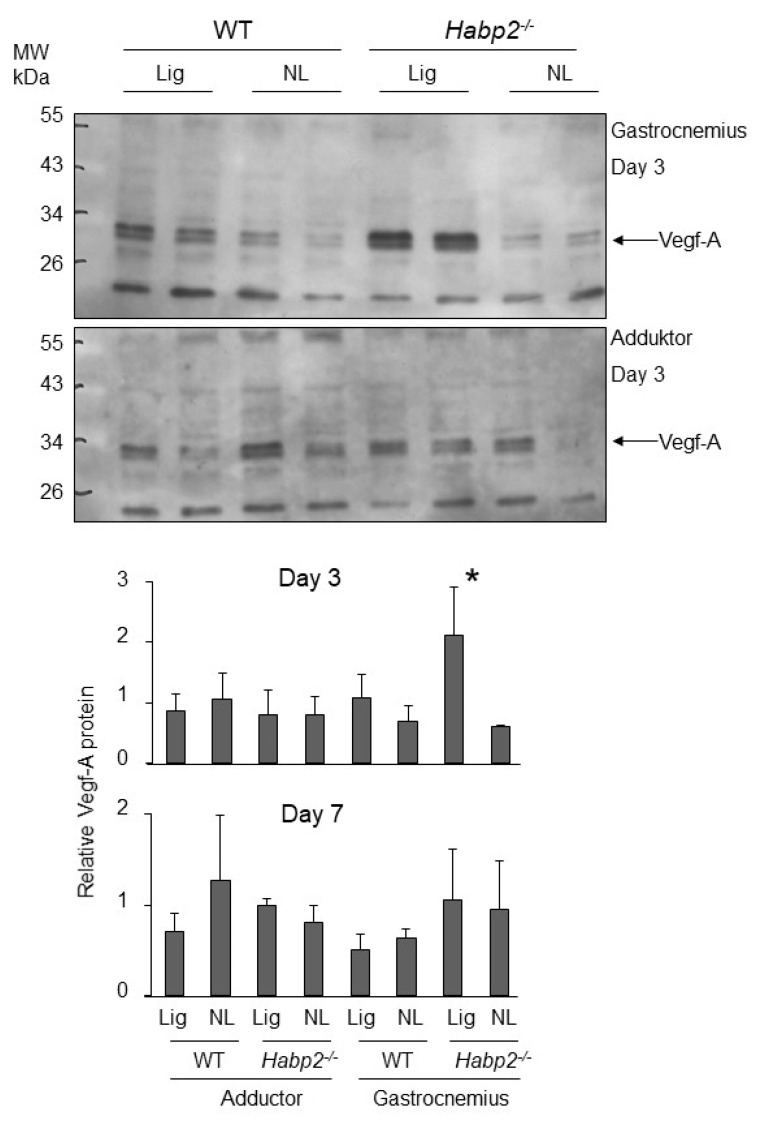
Changes in VEGF protein levels in the *Habp2*^-/-^ mice after hind limb ischemia: In the hind limb muscles of WT- and *Habp2^-/-^* mice, VEGF-A protein was detected by western blotting at day 3 in the gastrocnemius muscle (top panel) and adductor muscles (bottom panel). VEGF-A was normalized to the expression of cytochrome C oxidase, as measured by western blotting on stripped blots. Relative VEGF-A levels were quantified by densitometry (means + SEM, *n* = 4, * *p* < 0.05).

*VEGF expression in the Habp2^-/-^ mice subjected to hind limb ischemia:* We then examined whether the levels of the long forms of VEGF were higher in mice in the absence of endogenous FSAP (*Habp2^-/-^* mice). For this, we used the hind limb ischemia model where angiogenesis is induced in the gastrocnemius muscle after femoral artery ligation [16]. Although, collateral growth in the adductor muscle was enhanced in *Habp2^-/-^* mice, capillary density in the gastrocnemius muscle was not altered at 21 days. Western blotting showed that, at an earlier time point of day 3 after ligation, VEGF-A protein was significantly upregulated in the gastrocnemius muscle of *Habp2^-/-^* mice compared to WT mice, whereas no increase was observed in the adductor muscle (Figure 8). At day 7, after ligation, this effect on VEGF-A protein was no longer evident. The VEGF-A isoform detected was largely the VEGF_121_ dimer (30 kDa), and longer forms seemed not to be produced in this tissue. It was also possible that longer forms were produced and then cleaved to smaller forms. No change in VEGF-A mRNA was detected at the day 3 time point (Supplementary Figure S3). Thus, it was not possible to establish a causal link between the absence of endogenous FSAP and the increased presence of longer forms of VEGF in this experimental system.

## 4. Discussion

VEGF_165_ was cleaved at position 124/125 in the heparin/neuropilin-binding region that resembles a classic, multi-basic, FSAP cleavage site. Because the disulphide bonds in this region were still intact, there was no difference in mobility of the protein under non-reducing conditions, only under reducing conditions. Only the larger forms of VEGF, 165 but not 121, bound to FSAP, thus conferring specificity to the process. FSAP also bound to neuropilin but not to VEGF-R2, and partially inhibited VEGF_165_ binding to neuropilin. The binding and cleavage by FSAP was more pronounced in the presence of heparin, which is cofactor for FSAP. As previously reported, plasmin cleaved VEGF after position 110, thereby completely separating the receptor-binding domain from the heparin/neuropilin-binding domain [33,34]. Cleavage in the C-terminal region of VEGF_165_ occurs naturally in vivo, and such cleaved forms have been observed in tumor ascites [35] as well as wound fluids [34]. Such cleavage is likely to reduce the association of VEGF long forms with the matrix and increase their mobility, thus raising the possibility of activating VEGF receptor as well as modulating their binding to co-receptors [27]. 

In proliferation, migration, and signal transduction assays on HUVEC, no consequence of the VEGF_165_ cleavage was observed. This cleavage did not affect the induction of proliferation of VEGFR-transfected BAF3 cells either. This can be explained by the fact that the position of the cleavage in VEGF_165_, in relation to the disulphide bond, was such that the molecule was still held together and preserved some of its characteristics. Other proteases such as urokinase, matrix metalloprotease (MMP), elastase, and tissue kallikrein also cleave the long forms of VEGF in this region and modulate its activity in different ways depending on the site of cleavage.

Matrigel-embedded growth factors provide a model to study neovascularization in vivo [29]. Growth factor-reduced matrigel is the matrix of mouse sarcoma, which contains 1851 unique proteins including classical matrix proteins such as laminin, collagen IV, entactin/nidogen, fibronectin, and heparan sulphate proteoglycans [36]. Heparin increases angiogenesis in this model by multiple mechanisms that probably include modifying growth factor’s presentation to the cellular receptors [37]. In this model, FSAP inhibited the activity of VEGF_165_, bFGF, and a combination of both, and microvascular density was decreased. We used BS1 and vWF as markers for endothelial cells and α-SMC actin as a marker for pericytes/VSMC. Individually, the staining of each marker was reduced by FSAP, indicating that FSAP directly inhibited both cell types. FSAP may also influence paracrine interactions between them, potentially, leading to the same end result. In this model, bFGF and VEGF_165_ were known to upregulate PDGF-BB, and this in turn may have been responsible for the recruitment of the smooth muscle cells [38]. Smooth muscle cells and pericytes can indirectly contribute to the regulation of neo-vascularization through various paracrine/juxtacrine mechanisms [39], and we have previously shown that FSAP can inhibit PDGF-BB [18]. The inhibitory effect of FSAP on bFGF may be related to a complex interaction between FSAP and bFGF, as described before [19]. This result also illustrates the fact that the inhibitory effect of FSAP in this model was not limited to VEGF_165_, and a completely different mechanism of action, independent of the growth factor, cannot be excluded. 

In view of the lack of the effect of FSAP on VEGF-mediated proliferation and migration of endothelial cells in vitro*,* the strong reduction of angiogenesis in the matrigel model was remarkable. Because FSAP cleavage of VEGF_165_ led to a VEGF_121_-like molecule, the difference in the in vitro and in vivo results must be related to the difference between the two isoforms. It was possible that in the matrigel model, the VEGF was presented to the cells in a form that was bound to the matrix, and that the haptotaxis-effect was altered by FSAP. Evidence for proteolysis-mediated modulation of haptotaxis of matrix-anchored VEGF has been demonstrated before [40]. The cleavage of VEGF by FSAP may also alter the sequestration of the growth factor by the matrix or its spatial complexity, both of which are known to be important for VEGF activity [41]. VEGF can also activate leukocytes and other VEGFR-bearing cells, which may indirectly mediate the inhibition of angiogenesis by FSAP. An effect of VEGF in the matrigel model through changes in vascular permeability was also a possibility. The time course of the in vitro experiments, ranging from minutes to 24 h, were different from the in vivo matrigel experiments that lasted a few days, and this could also account for the differences in the results. 

The induction of ischemia led to angiogenesis in the gastrocnemius muscle in the hind limb ischemia model. Mice without endogenous FSAP showed no changes in their angiogenesis response in this model, indicating that endogenous FSAP was not involved in this process [16]. However, collateral vessel growth was increased in the adductor muscle. Application of exogenous FSAP, directly into the adductor muscle, decreased collateral growth there, but increased angiogenesis in the gastrocnemius muscle. This was most likely a response to the decreased collateral growth in the vessels feeding the gastrocnemius muscle [16]. In the neointimal growth model, exogenous FSAP decreased neointima formation [15] and a lack of endogenous FSAP increased it [18]. Thus, in two independent models of vascular growth and repair, FSAP seemed to be involved in regulating vascular remodeling but not angiogenesis. This also fits with the lack of difference in neovascularization in *Habp2^-/-^* mice in the matrigel model (see Table 1 for an overview). 

We chose to study the hind limb ischemia model to investigate processing of long forms of VEGF because we have performed studies using this model in FSAP-deficient mice [16]. However, we did not detect any VEGF_165_ in this tissue and it was not possible to find any direct evidence for changes in cleavage of this VEGF isoform. However, we did observe an increase in VEGF_121_ in *Habp2^-/-^* in the absence of any increase in mRNA. Further experiments would be required to study VEGF isoform-specific mRNA and protein to provide compelling evidence for this hypothesis. In this model system there was an upregulation of uPA and MMP-9 at the mRNA and protein level in the gastrocnemius muscle, which could also account for the fact that only the short form of VEGF was detected [16]. Such experiments would be more conclusive in performed in relation to angiogenesis in the eye or the brain because they display the sharpest concentration gradients of VEGF_165_ [27].

Although endogenous FSAP did not regulate angiogenesis and neovascularization, it did influence remodeling of vessels in general. VEGF_165_ cleavage, binding properties towards neuropilin, and angiogenesis in matrigel were inhibited by exogenous FSAP. Thus, FSAP has the potential to modulate VEGF_165_-mediated angiogenesis that may be relevant in some pathophysiological conditions.

## Figures and Tables

**Table 1:** Summary of the effects of FSAP on vascular growth and repair processes.

	*Habp2^-/-^* Mice: Lack of Endogenous FSAP	Local Application of Exogenous Purified FSAP
Neointima formation in response to intraluminal injury in the femoral artery	Increased [15]	Decreased [18]
Femoral artery ligation and stimulation of collateral development in the adductor muscle	Increased [16]	Decreased [16]
Femoral artery ligation and stimulation of angiogenesis in the gastrocnemius muscle	No change [16]	Increased, probably as a consequence of decreased collateral growth [16]
Matrigel/growth factor model of angiogenesis	No change (this study)	Decreased (this study)

## Supplementary Materials

The following are available online at https://www.mdpi.com/2073-4409/8/11/1396/s1, Figure S1: Cleavage of VEGF_165_ and VEGF_121_ by FSAP, Figure S2: Microvascular density in matrigel plugs, Figure S3: *Vegf-A* mRNA in the hind limb ischemia model.
