# Supplementary material for: VEGF-A-Cleavage by FSAP and Inhibition of Neo-Vascularization
Source: Cells. 2019 Nov 6;8(11):1396. doi: 10.3390/cells8111396 (PMC6912458; doi:10.3390/cells8111396)
Supplement: Supplementary file 1 [file cells-08-01396-s001.pdf]

Supplementary Fig. S1

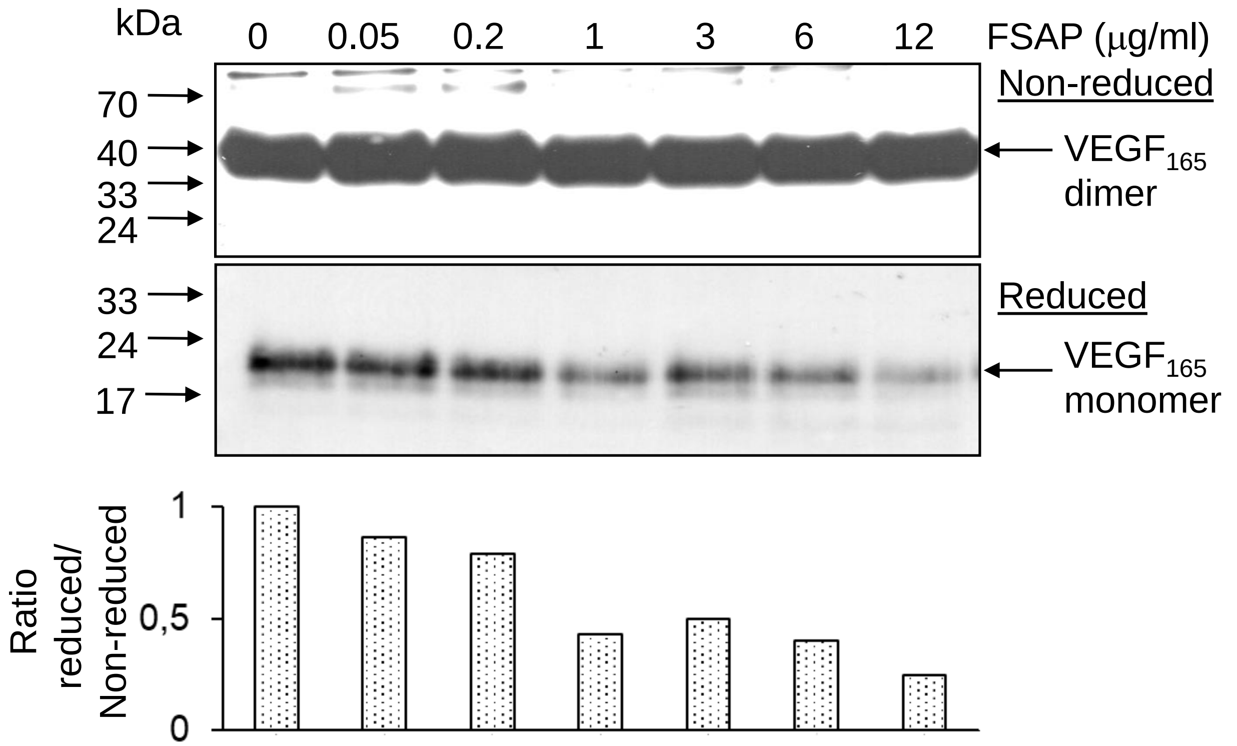

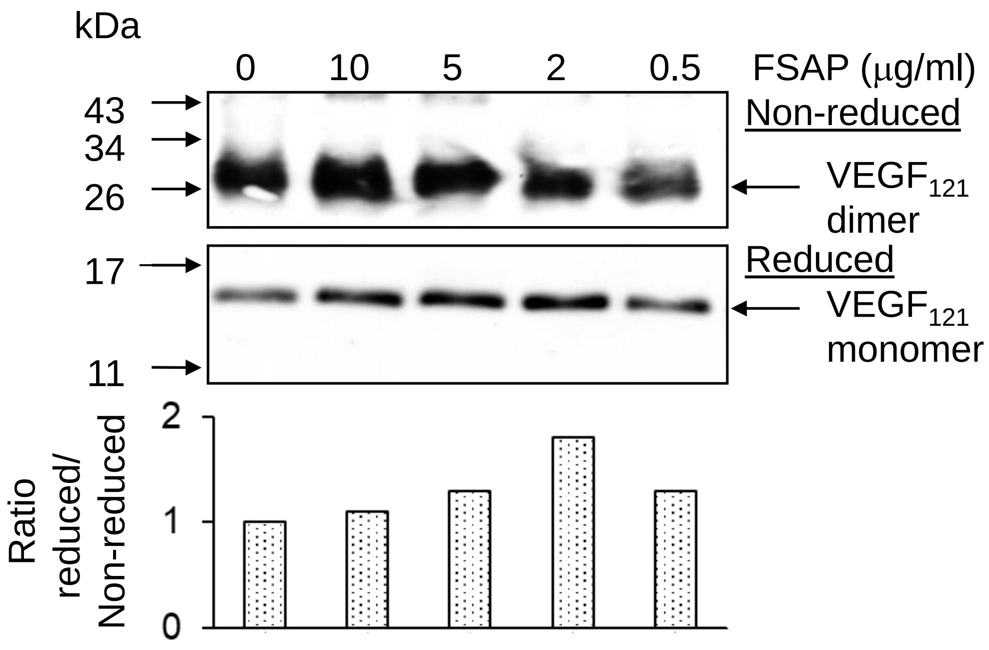

Cleavage of $VEGF_{165}$ and $VEGF_{121}$ by FSAP: Mixtures of FSAP (0-12 μg/ml) and $VEGF_{165}$ (top panels) or $VEGF_{121}$ (bottom panels) (1 μg/ml) were incubated for 1 h at 37°C and Western blotting was performed with an anti-VEGF antibody under non-reducing or reducing conditions. Ratio of reduced to non-reduced VEGF after densiometric analysis is presented in the bar charts.

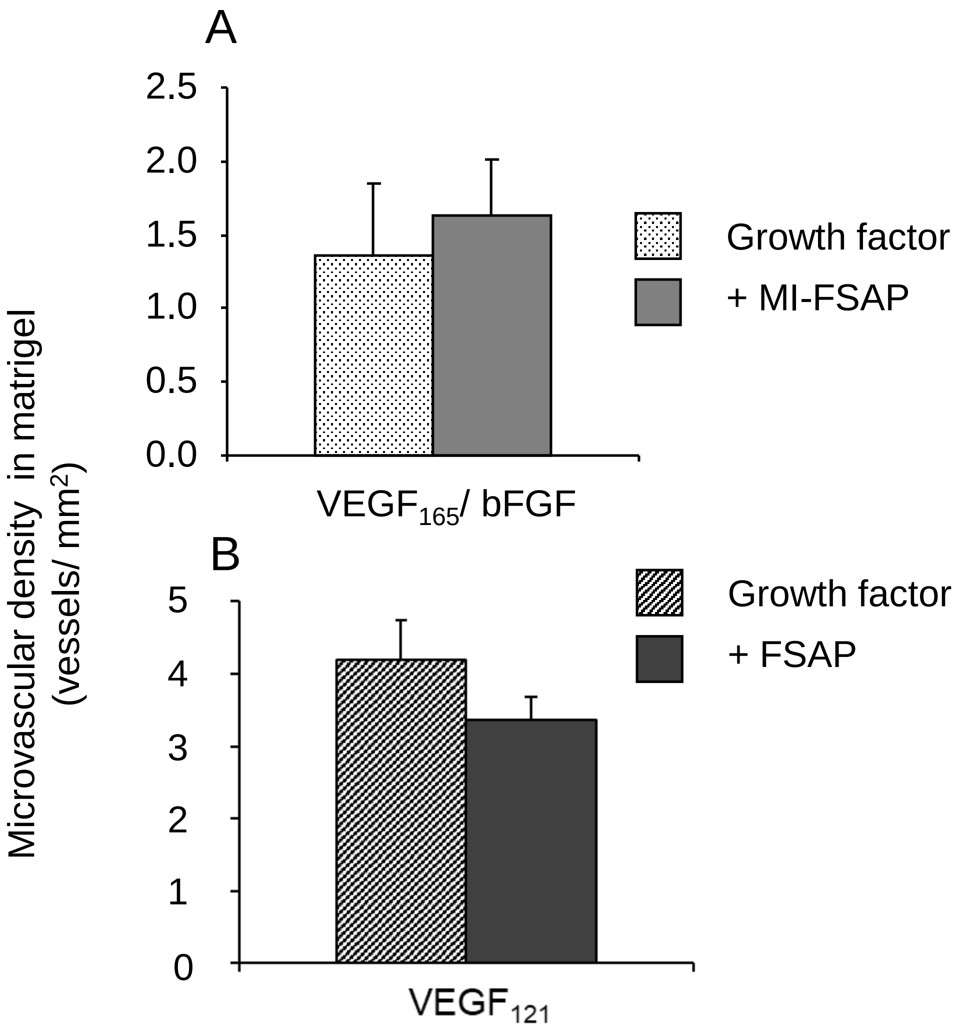

<u>Microvascular density in matrigel plugs:</u> A) Effect of MI-FSAP. Matrigel was supplemented with heparin (200 μg/ml) and either buffer or MI-FSAP (G534E- SNP) (12 μg/ml) as well as $VEGF_{165}$ and bFGF (200 ng/ml each) and applied subcutaneously into mice. After 7 days the plugs were removed, fixed and quantitative analysis of microvascular density was performed (n= 3 mice; mean $\pm$ SEM). B) Same as above except that $VEGF_{121}$ and FSAP was tested (n= 3 mice; mean $\pm$ SEM).

Supplementary Fig. S3

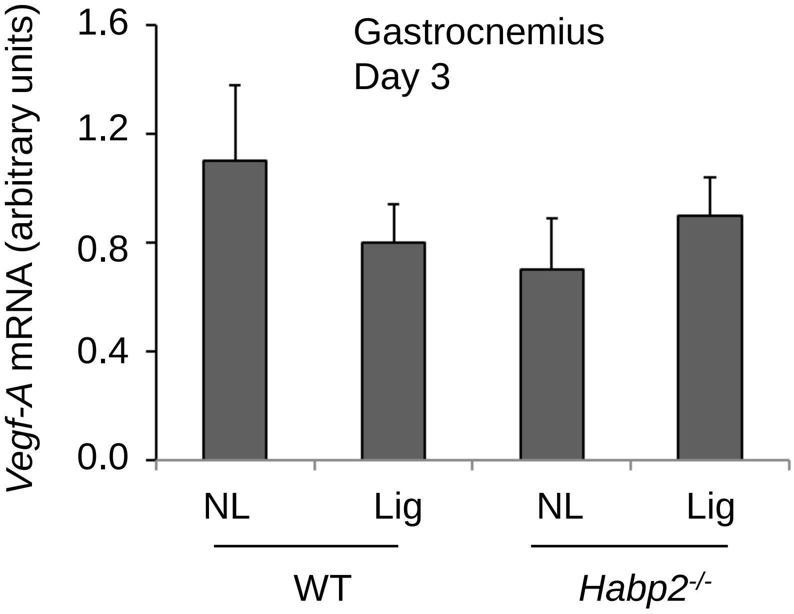

Vegf-A mRNA levels in the hind limb ischemia model: *Vegf-A mRNA* was analyzed from femoral arteries with ligation (Lig) or non ligated (NL) at day 3 in the gastrocnemius muscle. mRNA levels are shown, relative to *Gapdh,* as fold increase over non-ligated WT mice, mean ± SEM, n=4. Real-time quantitative PCR analysis was done as described in references 15 and 16 of the main manuscript.
